# Pathophysiology of encapsulating peritoneal sclerosis: lessons from findings of the past three decades in Japan

**DOI:** 10.1007/s10157-023-02360-y

**Published:** 2023-06-06

**Authors:** Masaaki Nakayama, Masanobu Miyazaki, Chieko Hamada, Yasuhiko Ito, Kazuho Honda, Peritoneal Biopsy Study Group of the Japanese Society for Peritoneal Dialysis

**Affiliations:** 1 St Luke’s International Hospital, Kidney Center, Akashi-cho 9-1, Chuo-ku, Tokyo, Japan; 2 Miyazaki Clinic, Nagasaki, Japan; 3 Juntendo University, Tokyo, Japan; 4 Aichi Medical University, Nagoya, Japan; 5 Showa University, Tokyo, Japan

**Keywords:** Encapsulating peritoneal sclerosis, Neo-membrane, Neutral solution, Peritoneal biopsy, Laparoscopy, Peritoneal histology, Ultra-fine endoscope

## Abstract

Encapsulating peritoneal sclerosis (EPS), a condition with a high mortality rate, is a serious complication of peritoneal dialysis (PD). In Japan, EPS became a central issue in the clinical setting during the mid-90s and the beginning of this century. However, following the introduction of biocompatible neutral PD solutions containing lower levels of glucose degradation products, the incidence and clinical severity of EPS has been greatly lessened. During the past three decades, the etiology of EPS has been elucidated by findings obtained by peritoneal biopsy, laparoscopy, and surgical intervention. Accumulating findings suggest the need for a paradigm change on the nature of EPS pathophysiology; notably, EPS appears not to reflect peritoneal sclerosis per se, but rather the formation of a neo-membrane as a biological reaction to peritoneal injury. This narrative review looks back on the history of EPS in Japan, and discusses EPS pathophysiology, the impact of neutral PD solution on peritoneal protection, and a future novel diagnostic approach, ultra-fine endoscope, for the identification of patients at high risk of EPS.

## Introduction

Encapsulating peritoneal sclerosis (EPS) is the most serious complication in peritoneal dialysis (PD) therapy, but this condition is about to become a disease of the past in Japan. The present narrative review discusses the pathophysiology of EPS, based on the findings in the past three decades in Japan, an interval that spanned the start and end of era of EPS. This discussion includes the role of neutral PD solution for peritoneal protection, and the use of a novel diagnostic approach employing PD-specific laparoscopy.

## Start of EPS in Japan

EPS is characterized by peritoneal encapsulation due to fibrosis, resulting in gastrointestinal obstruction [1, 2]. The common clinical symptoms of this condition include anorexia, nausea, vomiting, and weight loss, other presentations include hemoperitoneum and sterile recurrent peritonitis [1, 2]. EPS was a serious complication of PD, with a high associated mortality rate of more than 39–43% in Japan in the 1990s and early in the twenty-first century [3–5]. The first case of EPS was reported in 1985, 3 years after PD therapy was introduced in Japan. Thereafter, the number of EPS patients increased rapidly, with over-all incidence rising from 0.9% during the interval of 1980–1994 [3] to 2.5% in 1999–2003 [4]. The absolute number of patients on PD grew during the mid-90s; the accumulating number of EPS patients became a serious burden in the Japanese PD community [4] (Table 1).

**Table 1:** Incidence of encapsulating peritoneal sclerosis in Japan

Authors (Ref.)	Nomoto [3]	Hoshii [7]	Nakamoto [4]	Kawanishi [5]	Nakayama [50]
Survey	1980–1994 Adult	1981–1995 Children	1980–2000 Adult	1999–2003 Adult	2008–1012 Adult
Solution (glucose) type	Acidic	Acidic	Acidic	Acidic	Neutral
EPS/PD population	62/6923	11/687	256/11,549	48/1958	14/1358
Overall incidence	0.9%	1.6%	1.7%	2.5%	1.0%
PD duration and incidence	N/A	6.6% (> 5 yrs) 12.0% (> 8 yrs)	N/A	0% (< 3 yrs) 0.7% (< 5 yrs) 2.1% (< 8 yrs) 5.9% (< 10 yrs) 5.8% (< 15 yrs) 17.2% (≥ 15 yrs)	0.3% (< 3 yrs) 0.6% (< 5 yrs) 2.3% (< 8 yrs) 1.2% (≥ 8 yrs)
PD duration at EPS (average)	65.4 months	115.2 months	99.6 months	114.3 months	67.3 months
No peritonitis history among EPS	8.0%	18.2%	4.3%	21.0%	42.8%
Mortality rate	43.5%	18.2%	39.1% (2 years)	37.5%	14.3%
Therapy corticosteroid/enterolysis	NA/14.5%	9.1%/18.2%	83.2%/30.7%	38.5%/58.3%	85.7%/14.3%

*N/A* not available

**Fig. 1:**
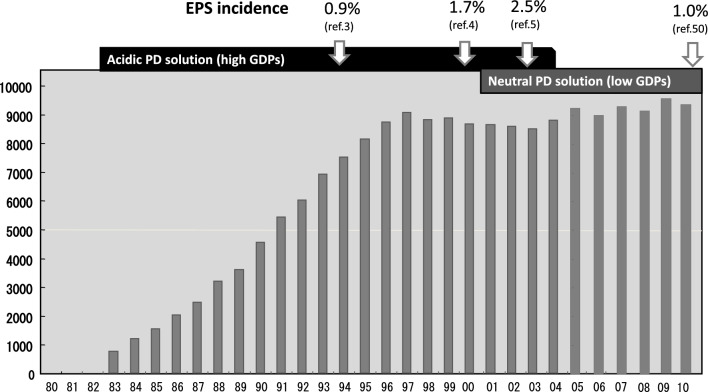
Number of peritoneal dialysis (PD) patients and incidence of encapsulating peritoneal sclerosis (EPS) in Japan. The Working Group on Sclerosing Encapsulating Peritonitis (SEP) of the Ministry of Health, Labour and Welfare of Japan, issued a draft clinical guide for the diagnosis and management of SEP in 1998 [56]. Thereafter, an ad hoc committee of the International Society of Peritoneal Dialysis (ISPD) published a position statement on the diagnosis and management of EPS in 2000 [2], and a Japanese working group issued a proposal for the diagnosis and treatment of EPS in 2005 [57]. The Japanese Society for Dialysis Therapy (JSDT) issued a guideline for preventing EPS in 2009 [58], which recommended a planned PD withdrawal in those patients on long-term PD therapy who present a persistent high transport state

The impact of EPS was large, reflecting the concomitant rising number of PD population in Japan. The penetration of PD increased rapidly during 80s and 90s (until 1997); however, it subsequently stagnated due to the EPS shock to medical personnel until recently (Fig. 1).

EPS was a serious problem for Japanese PD experts, given the condition’s high mortality rate and the lack of an established treatment policy [2]. However, the problem of EPS in Japan remained a suspicion for experts in other countries, as shown by the fact that a presenter at the “pros and cons” session at the Ninth Congress of the International Society for Peritoneal Dialysis (held in 2001 in Montreal, Canada) was asked “whether the high incidence of EPS in Japan [was] the result of over-diagnosis” [6]. This distinction raised the question of what differed between Japan and other countries in the practice of PD. Examination of the clinical use of PD in Japan revealed several aspects of the practice that were specific to Japan at that time, including the following: (1) a very low rate of peritonitis, (2) better patient survival due to fewer cardiovascular comorbidities, and (3) the very low frequency of renal transplantation. As a result, PD patients in Japan typically underwent PD therapy for relatively longer durations than patients in other countries; it was not uncommon for Japanese patients to be maintained on PD for more than 10 years. This long-term use of PD was strongly associated with the primary pathology of EPS [5–9].

## Pathophysiology

### Classical hypothesis: EPS as a severe form of peritoneal sclerosis (PS)

**Fig. 2:**
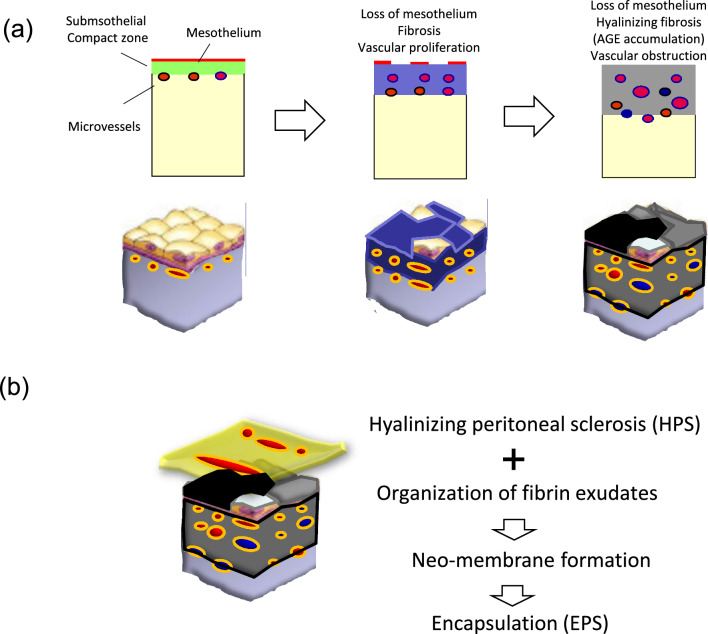
Histological schema of peritoneal dialysis (PD)-associated hyalinizing peritoneal sclerosis (HPS) (**a**) and encapsulating peritoneal sclerosis (EPS) (**b**). Processes of PD-associated hyalinizing peritoneal sclerosis (HPS) (**a**). Normal peritoneum is covered by mesothelial monolayer and supported by thin submesothelial compact zone. Microvessels are sparsely distributed. After PD using conventional acidic PD solution with high GDPs, the surface mesothelial cells are detached partially. The submesothelial compact zone becomes thick with fibrosis. Peritoneal microvessels are proliferated by angiogenic stimuli of the bio-incompatible PD solutions. After long-term PD using conventional PD solutions, the mesothelial cells are completely lost and the submesothelial compact zone becomes thicker with extensive fibrosis with hyalinosis degeneration of collagen fibers (hyalinizing peritoneal fibrosis). The wall of the microvessels, especially at post-capillary venules, shows hyalininous thickening with luminal narrowing or obstruction (hyalinizing vasculopathy). The state of advanced peritoneal sclerosis induced by PD is called hyalinizing peritoneal sclerosis (HPS), distinguished from EPS. Processes of encapsulating peritoneal sclerosis (EPS) (**b**). In the era of the conventional PD solution, EPS occurred in the background of HPS. The increased peritoneal permeability induces fibrin exudation on the surface of the peritoneum, but the exudates are washed out by undergoing PD treatment. If the PD treatment is discontinued, then the fibrin exudate become organized and forms neo-membrane on the surface of the proper peritoneum. The organized neo-membrane promotes adhesion and encapsulation of the intestines and finally developed to EPS

The exact mechanism by which EPS develops remains unclear. However, it is generally assumed that EPS results from functional changes of the peritoneum during the course of long-term PD [10, 11]. Given that the incidence of EPS increases with the duration of PD, it was speculated that changes in the peritoneum may play a central role in the development of this condition. Peritoneal sclerosis (PS) is a uniform change of the peritoneal membrane that is associated with PD treatment [10]. PS is characterized by a loss of mesothelium and a progressive thickening of the sub-mesothelial layer as PD duration prolonged [12]. In addition, PD therapy also characteristically is associated with the progression of vasculopathy, a thickening of the vascular walls, as well as luminal obstruction at the level of the post-capillary venule [12] (Fig. 2a).

Therefore, it was speculated that the pathophysiology of EPS represents a severe form of PS. However, histological studies failed to confirm this hypothesis. Notably, three separate studies in Japan [13–15] did not detect differences in the thickness or vascular density of the peritoneum when comparing between patients with EPS and controls (Table 2). These results indicated that EPS may not simply represent a severe form of PS.

**Table 2:** Comparisons of peritoneal histology between encapsulating peritoneal sclerosis (EPS) and non-EPS

Authors (ref.)	Sherif et al. [13] EPS [12] vs non-EPS [23]	Tawada et al. [14] EPS (*n* = 10) vs non-EPS (*n* = 73)	Honda et al. [15] EPS (*n* = 10) Non-EPS (*n* = 52)
Mesothelial detachment	NS	NS	N/A
Sub-mesothelial compact zone thickness	NS	NS	NS
Vascular density	NS	NS	NS
Vasculopathy	NS	NS	NS
Neo-membrane formation	NS	NS	Significant
Fibrin stain	Significant	Significant	N/A

*NS* not significant; *N/A* not available

### Novel hypothesis: formation of neo-membrane and EPS

**Fig. 3:**
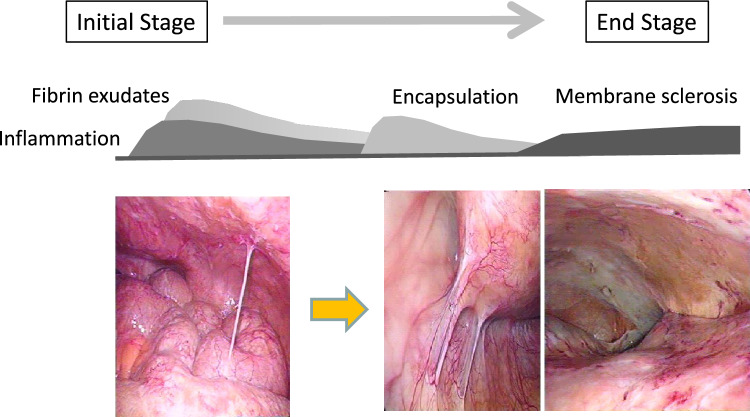
Progression of encapsulating peritoneal sclerosis (EPS) and laparoscopic findings of peritoneal membrane

What, then, are the essential characteristics of EPS? Laparoscopic examination provided valuable answers to this question. Typically, the clinical course of EPS is self-limiting [16, 17]. In the initial stages of EPS, there are signs of exudates, ascites, and vascularization. As the disease progresses, an encapsulating membrane extends over the intestine, Finally, in the end stage, a thick, dense membrane (“neo-membrane”) forms over the parietal wall and intestines (Fig. 3).

Histologically, this encapsulating neo-membrane, which covers the original membrane, exhibits characteristics distinct from the proper membrane; typically, the neo-membrane is rich in fibrin and vessels [18], and so can be separated from the original peritoneal membrane [19]. One interpretation is that the essence of EPS is the formation of the neo-membrane.

Why does this neo-membrane form over the existing peritoneum during extended PD? The mechanism of this change remains unclear. However, there exist some hints to the pathophysiology of the neo-membrane. Notably, it has been reported that the macrophage profile in the peritoneum differs between patients with and without EPS [20]. Interestingly, the EPS peritoneum is infiltrated by macrophages, predominantly M2-type macrophages [20], indicating that neo-membrane formation may be associated with a physiological reaction in response to peritoneal injury typically emerged in wound healing [21].

Therefore, neo-membrane formation is an inflammatory response of the peritoneum induced by PD, which induces fibrin exudate from hyperpermeable peritoneal vessels, triggering biological reactions that causes fibrosis and adhesion between the neo-membrane and the peritoneum per se; this adhesion leads in turn to the restriction of intestinal mobility and finally results in EPS (Fig. 2b).

## Peritoneal damage by acidic PD solution

**Fig. 4:**
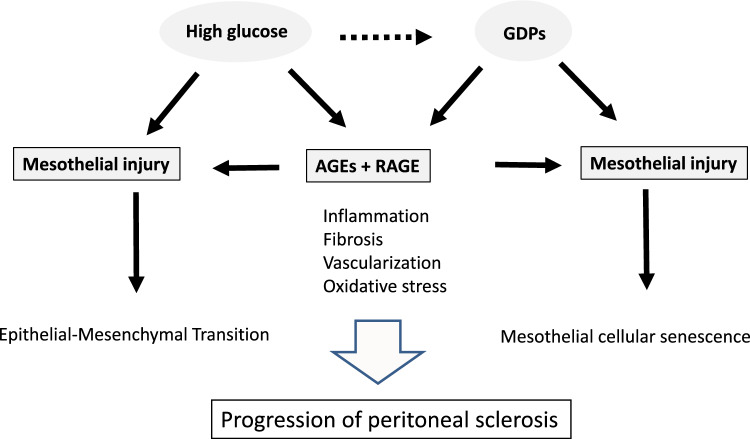
Pathogenesis of peritoneal sclerosis (PS) induced by conventional acidic peritoneal dialysis (PD) solutions containing high levels of glucose and glucose degradation products (GDPs). *AGE* advanced glycation endproduct; *RAGE* receptor for AGEs; this receptor is present in mesothelial cells, endothelial cells, and myofibroblasts

What factors are the primary inducers of peritoneal damage in PD therapy? Given that EPS develops even in patients who never experienced peritonitis (Table 1), it was suspected that the PD solution itself may play a role in the development of peritoneal damage. In the 90s, special attention was paid to a possible contribution by GDPs. A study in Sweden revealed that heat-sterilized PD solutions permit lower cellular proliferation than do filter-sterilized solutions [22], indicating that some toxic products are being generated during the heat-sterilization process. Subsequent work revealed that the toxic molecules were GDPs, including 5-(hydroxymethyl)furfural, formaldehyde, furaldehyde, acetaldehyde, and dicarbonyl compounds like 3-deoxyglucosone, glyoxal, and methylglyoxal [23]. In other work, these GDPs have been shown to exhibit direct toxicity to the mesothelium [23].

On the other hand, the role of AGEs in peritoneal damage also has been a topic of discussion. AGEs are generated by non-enzymatic chemical reactions between proteins and glucose or carbonyl compounds. Clinically, AGEs are generated in the PD peritoneum following the initiation of PD, as was first reported in human peritoneum from Japan [24]. Additionally, AGEs accumulate as a function of PD duration, especially in proximity to the vascular wall, a process that is associated with an increased degree of solute transport [25, 26]. Furthermore, increased AGE content has been observed in peritoneal tissue from patients with EPS [27].

GDPs induce mesothelial injury, which triggers both the epithelial–mesenchymal transition and enlargement of the mesothelial surface area (a marker of senescence), leading to PS [28–30]. AGEs induce cellular injury by protein modification and collagen cross-linking, and may trigger pro-inflammatory conditions via binding to the receptor for AGE (RAGE) in peritoneal tissue (Fig. 4) [31, 32], especially in patients with EPS [33].

## Clinical impact of neutral PD solution with lower levels of GDPs

In response to the above findings, PD solutions with lower levels of GDPs (i.e., neutral PD solution) were developed in the expectation that such reagents would reduce toxicity compared to conventional (acidic) PD solutions.

At present, three kinds of neutral low-GDP PD solutions are available commercially around the world, including a lactate-based neutral solution, a bicarbonate-based solution, and a solution that incorporates both lactate and bicarbonate. In Japan, the lactate-type glucose-based neutral PD solution has been available from the start of this century, and since 2004, neutral PD solution has completely replaced the conventional acidic lactate PD solution. Furthermore, the mixed-type (bicarbonate and lactate) PD solution became available in the clinical setting since 2014.

The use of neutral PD solutions has been reported to result in increased levels of cancer antigen-125 (CA 125) and procollagen peptide, and decreased levels of interleukin (IL)-6 and hyaluronic acid in dialysate effluents [34]. These changes may be reflected by the improved viability of peritoneal cells such as mesothelial cells and fibroblasts, as well as the decreased pro-inflammatory condition in the abdominal cavity. However, the effects of neutral PD solution on the peritoneal membrane function have been controversial, and only one study (the balANZ trial) has reported a stable membrane transport state over 24 months [35]. In Japan, a 15-month observational study reported that the use of neutral PD solution provided an improvement in peritoneal function in those patients with conventional PD solution who initially presented with a higher transport state, accompanied by a significant decrease in matrix metalloprotease-2 (MMP2) levels in the effluent [36]. These results indicated that the neutral PD solution ameliorates pro-inflammatory conditions, normalizing the membrane transport state. According to the annual cross-sectional survey conducted by the Japanese Society for Dialysis Therapy (JSDT), no changes in peritoneal function by PD duration were observed in data obtained following the implementation of neutral PD solutions (Table 3) [37–40].

**Table 3:** Peritoneal function (Peritoneal Equilibration Test) and PD duration (data from JSDT registry)

PD duration (yrs)	< 1	> 1	> 2	> 4	> 6	> 8
Survey at 2016						
*N*	460	674	790	355	130	110
PET average (SD)	0.65 ± 0.15	0.68 ± 0.14	0.67 ± 0.13	0.65 ± 0.13	0.65 ± 0.11	0.63 ± 0.17
% High transporter	16.1%	18.8%	15.6%	8.5%	6.2%	13.6%
2015						
*N*	467	592	781	333	140	100
PET average (SD)	0.66 ± 0.14	0.68 ± 0.14	0.67 ± 0.12	0.65 ± 0.13	0.64 ± 0.11	0.62 ± 0.14
% High transporter	16.1%	17.1%	13.6%	10.8%	8.6%	7.0%
2011						
*N*	416	428	719	680		226
PET average (SD)	0.66 ± 0.14	0.67 ± 0.14	0.66 ± 0.13	0.63 ± 0.12		0.60 ± 0.13
% High transporter	13.9%	18.2%	14.9%	7.4%		7.1%
2010						
*N*	373	418	664	551		198
PET average (SD)	0.65 ± 0.15	0.67 ± 0.14	0.66 ± 0.13	0.64 ± 0.14		0.61 ± 0.14
% High transporter	17.4%	16.7%	13.4%	10.5%		7.6%

*SD* standard deviation

Reproduced from Annual Dialysis Data Report, The annual survey of Japanese Society for Dialysis Therapy Renal Data Registry; [37–40]

**Fig. 5:**
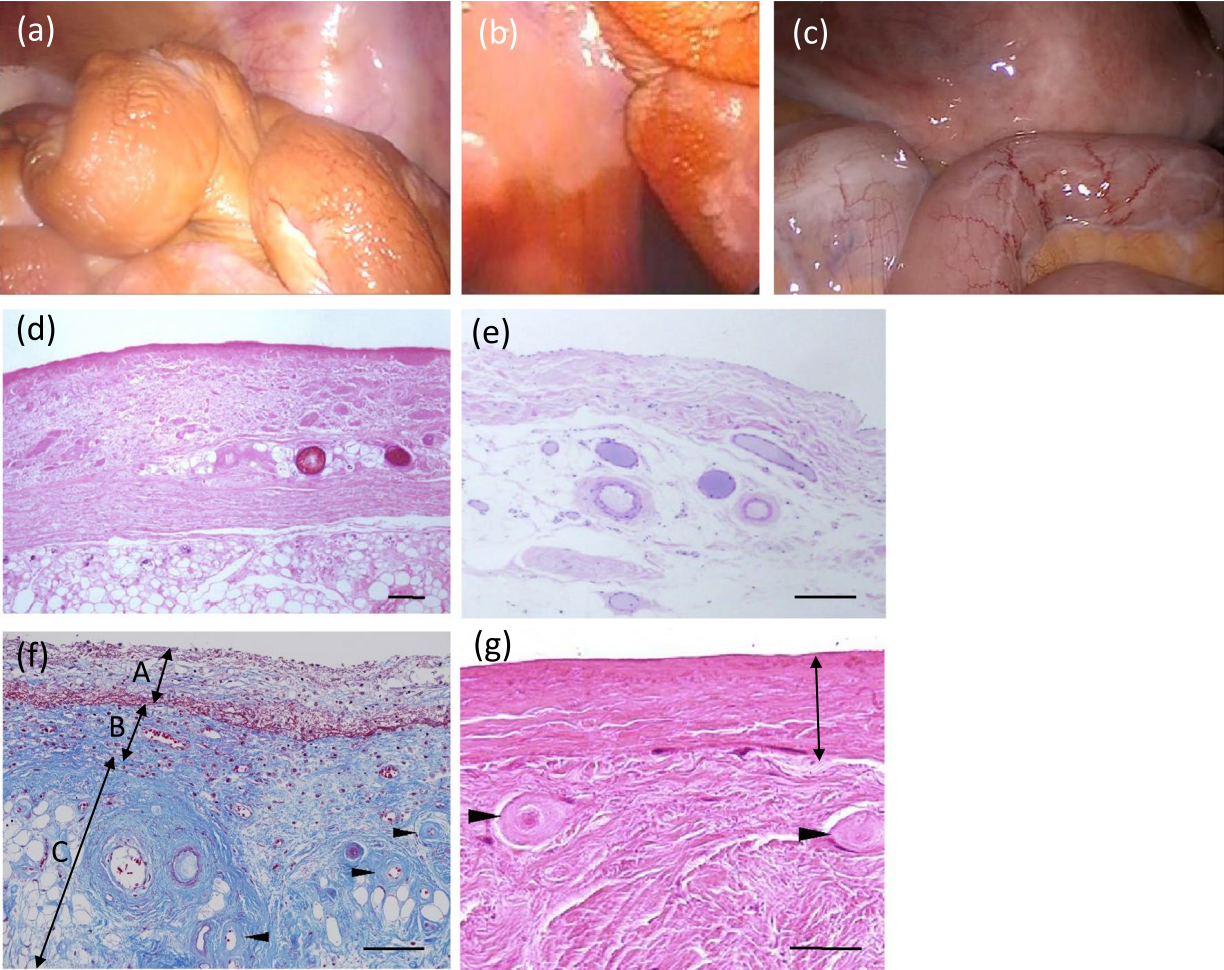
Representative macroscopic and microscopic pathology of the peritoneum undergoing long-term peritoneal dialysis (PD). Macroscopic findings by laparoscopy in a case undergoing PD for 12-years using conventional acidic PD solution (**a**), 6-years using acidic PD solution (**b**), or for 5.5-years using neutral PD solution (**c**). Histology of the peritoneum of the patient undergoing PD more than 10-years using conventional PD solution (**d**–**g**), or neutral PD solution (**e**) [**d**, **e**, **g** Hematoxylin and eosin stain, **f** Masson trichrome stain, **d**–**f** scale bar 100 μm, **g** scale bar 50 μm]. The image (**d**) shows a thickened compact zone with hyalinosis, complete loss of the mesothelial layer, fibrin exudates on the peritoneal surface, and a thickened vascular wall accompanied by luminal obstruction. The image (**e**) shows a preserved mesothelial layer and mild fibrosis of the compact zone without hyalinous degeneration of collagen fibers. No vascular wall thickening or obstruction are seen. The image (**e**) shows double-layered neo-membrane covering the proper peritoneum of omentum (autopsy). Superficial neo-membrane (A) contains fresh fibrin exudates and inflammatory cells due to accompanying peritonitis. Deeper neo-membrane (B) contains organization of fibrin exudates with microvascular proliferation. Proper peritoneum (C) shows fibrosis and hyalinizing vasculopathy of the post-capillary venules with luminal narrowing or obliteration (arrow heads). The image (**g**) shows neo-membrane (arrow) covering the surface of proper parietal peritoneum with extensive fibrosis and obstructive vasculopathy (arrow heads). Image (**e**) was kindly provided by Dr. Ishibashi of The University of Tokyo/Japanese Red Cross Medical Center

Regarding the morphological changes of the peritoneum, direct observation by laparoscopy, and histologic examinations by peritoneal biopsy, have been reported in Japan [41–48]. Laparoscopic examination of the peritoneum has provided multiple interesting findings. In the era of conventional PD solution, brownish coloration of the intestine and abdominal wall, considered a marker for the generation of AGEs, was common in patients undergoing PD (Fig. 5a). In contrast, such color change is not seen in patients using neutral solution; and these tissues instead look a healthy and lack AGE accumulations, even in patients undergoing long-term PD therapy (Fig. 5b).

These observations were validated histologically by comparing biopsies of parietal peritoneal samples obtained from patients undergoing PD using conventional solution or neutral solution from the initiation of PD [46]. Peritoneal thickening was observed in both groups, with no significant differences between patients maintained on conventional or neutral PD solutions. However, in contrast to the cases with conventional PD solution, patients using a neutral PD solution did not exhibit any relationship between vasculopathy levels and PD durations; amazingly, no patients treated with the neutral solution exhibited a complete vascular obstruction.

Based on the findings obtained from the patients using neutral PD solution, it appears that a lactate-based low-GDP neutral PD solution prevents the development of PS that was associated with the duration of PD treatment in the patients treated using conventional PD solutions [47–49]. To illustrate these results, a representative peritoneal histology is shown in Fig. 5c, e.

## The end of EPS struggle in Japan

Can the use of neutral PD solution actually suppress the development of EPS? To date (to our knowledge), no such well-controlled randomized study (RCT) has been conducted. Such a study would be impractical, because the incidence of EPS is very low, and the development of EPS takes a long time. In addition, from the perspective of experimental ethics, it would be difficult to justify such an RCT study. Instead, we conducted a prospective observational study (the NEXT-PD study) among patients who had been treated using a neutral solution since their initiation on PD [50]. A total of 1358 prevalent PD patients was recruited from 55 representative PD centers across Japan. During the observation period of an average of 3.3 years, 702 patients stopped PD; among these subjects, 153 died, and 549 patients were switched to hemodialysis (HD). Of these patients, three developed EPS. Among the 700 patients who remained on PD, 11 cases developed EPS. As a result, a total of 14 cases developed EPS during the study period, with an overall incidence of 1.0%.

Interestingly, increased EPS incidence by PD duration was not observed in this cohort (Table 1). In addition, better clinical outcomes of EPS were seen in the NEXT-PD study compared to previous studies in patients undergoing PD with conventional solutions [3–5] (Table 1). Together, these results indicated that the clinical severity of EPS was greatly ameliorated in the patients maintained on neutral PD solution, compared to the severity in patients maintained on conventional PD solution.

According to data collected as part of the annual survey conducted by the Japanese Society for Dialysis Therapy (JSDT)  [37], the prevalence of EPS among patients undergoing PD therapy was 4.5% (469 cases/10,505 total) at 2010. The prevalences in the groups were as follows: 0.5% in patients maintained primarily on PD (*n* = 3713), 8.3% in patients maintained primarily on HD (*n* = 5269), and 1.1% in patients maintained on combination therapy (HD + PD; *n* = 1053). With regards to the duration of PD among these patients, the prevalences of EPS were as follows: 0.4% in cases with less than 1 year of PD, 0.6% in cases with 1–2 years of PD, 0.7% in cases with 2–4 years of PD, 1.1% in cases with 4–8 years of PD, and 1.5% in cases with more than 8 years of PD. The relatively high prevalence of EPS among patients undergoing HD may reflect the improved survival of patients with EPS, and be a result of the use of corticosteroid therapy [51] in association with surgical enterolysis [19]. Among 696 patients who were diagnosed with EPS, 86.6% of the cases were treated by corticosteroids, and 77.7% of the cases received surgical enterolysis. Among all mortalities in Japan in 2011 (*n* = 28,730) for which a cause of death was reported, only 12 deaths were attributed to EPS; thus, the rate of death attributable to EPS was 0.04% in 2011 in Japan.

## In the era of neutral PD solution

In the 2019 PD guidelines of the JSDT, the regular use of the peritoneal equilibration test (PET) is recommended; PD needs to be stopped in cases with a long-standing high-transport state [52], given that such a state may indicate a severe form of PS. These recommendations reflected the elevated incidence of EPS seen with acidic PD solutions [53]. However, for cases treated using a neutral PD solution, the induction of peritoneal damage by the PD solution appears to have been lessened. Instead, peritonitis is now considered a risk factor for EPS [54].

Despite this progress, there remains the question of how to identify patients undergoing PD who are at elevated risk of EPS; this issue will continue to be a challenge for the practice of PD in Japan. The most reliable technique to confirm early-stage EPS is to inspect the peritoneal cavity by laparoscopy. However, current laparoscopic examination remain invasive, and cannot be performed repeatedly in patients undergoing PD.

**Fig. 6:**
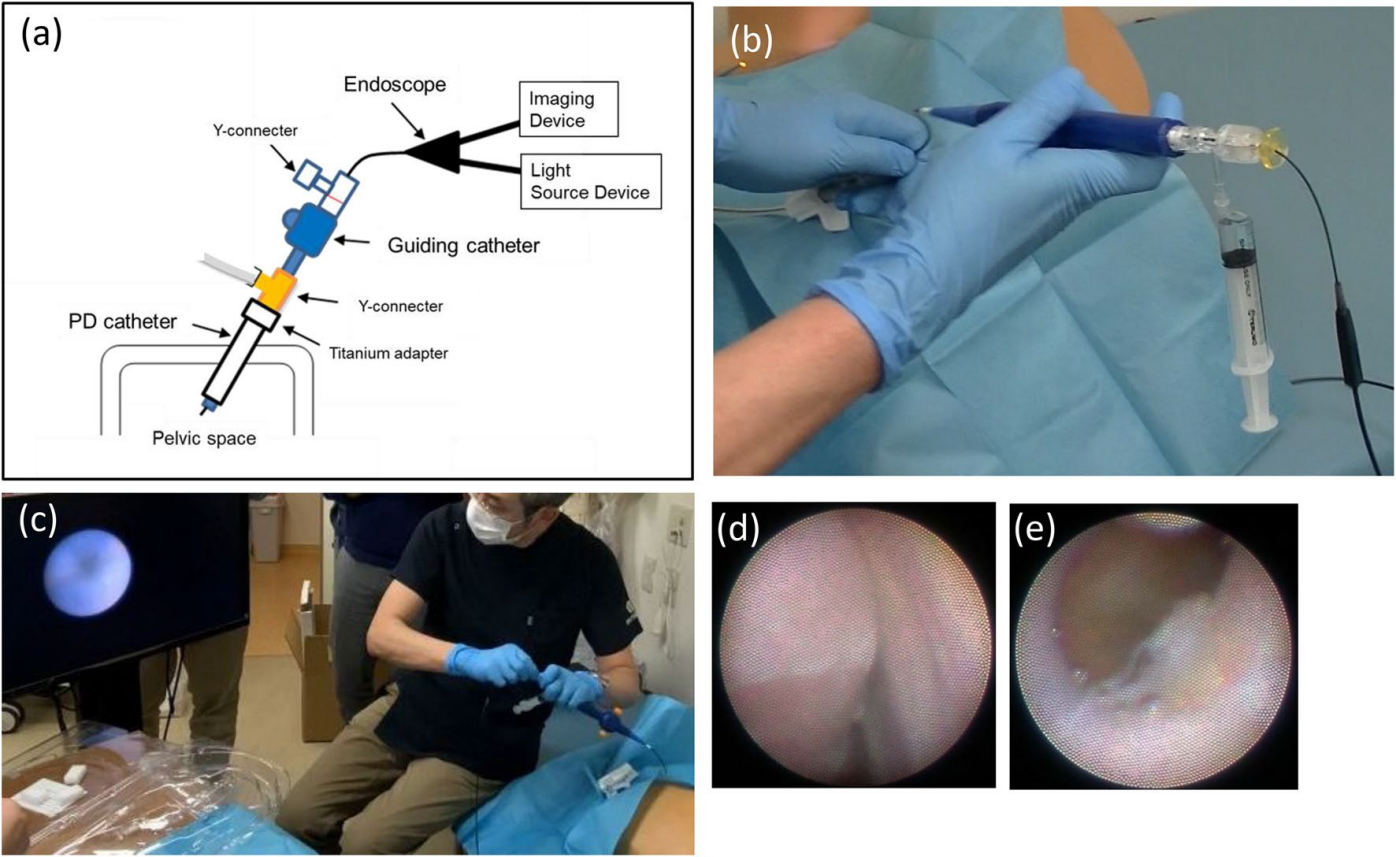
Overview of ultra-fine endoscopy developed specifically for use in patients undergoing peritoneal dialysis (PD). Outlines of laparoscopic examination using an ultra-fine endoscope (**a**–**c**), and laparoscopic findings in the peritoneum of patients undergoing PD. Note the presence of a localized fibrin net on the intestinal surface (**d**), and the cotton-like appearance of fibrin on the peritoneum (**e**). Images in these figures were kindly provided by Juntendo University School of Medicine

To address this challenge, an ultra-fine endoscope designed specifically for use in patients with PD was developed [55]. This endoscope has a diameter of 1.5 mm, allowing insertion into the PD catheter. Using a guiding catheter that bends the catheter tip, a 150° field can be viewed from the catheter tip (Fig. 6). A clinical trial in 10 patients demonstrated the safety of procedures performed using this equipment; such an instrument renders it possible to observe the peritoneal status of patients. Repeated endoscopic examinations are expected to reveal temporal changes in the peritoneal status of these cases during the course of PD therapy, and might provide further insights into the pathophysiology of the peritoneum (including EPS) in patients undergoing PD.

## Summary and conclusions

Based on the clinical experiences in Japan, conventional acidic PD solutions were hypothesized to be primary drivers of peritoneal membrane damage in patients undergoing PD therapy. The presence of GDPs in such PD solutions, and the associated production of AGEs in the peritoneal membrane, are thought to play a major role in the etiology of peritoneal damage (PS) in such cases. Empirical results, epidemiology, and histological analysis all indicate that low-GDP neutral PD solutions help to preserve peritoneal membrane integrity during PD, thereby lessening the risk of development of EPS.

One hypothesis has been considered that EPS represents a more severe form of PS. However, laparoscopic and histological findings suggested the need for a paradigm change regarding the pathophysiology of EPS. We conjecture that EPS is not, in fact, a form of PS, but is, instead a physiological wound-healing reaction to peritoneal injury. Endoscopic examination using an ultra-fine endoscope designed for the use in PD patients is expected to reveal temporal changes in the peritoneum during the course of PD therapy. This technique also may provide further insights into the pathophysiology of the peritoneum, including EPS, in patients undergoing PD.
